# Canine Leishmaniasis in Brazil: Serological Follow-Up of a Dog Population in an Endemic Area of American Visceral Leishmaniasis

**DOI:** 10.1155/2009/680790

**Published:** 2010-01-13

**Authors:** Alba Valéria Machado da Silva, Adelzon Assis de Paula, Daniela de Pita Pereira, Reginaldo Peçanha Brazil, João Carlos Araujo Carreira

**Affiliations:** ^1^Departamento de Protozoologia, FIOCRUZ, Rio de Janeiro, CEP 21040-360, Brazil; ^2^Centro de Ciências de Saúde, UFRJ, Rio de Janeiro, CEP 21941-901, Brazil; ^3^Laboratório de Bioquímica Molecular de Doenças Endêmicas, FIOCRUZ, Rio de Janeiro, CEP 21040-360, Brazil; ^4^Laboratório de Bioquímica e Fisiologia de Insetos, FIOCRUZ, Rio de Janeiro, CEP 21040-360, Brazil

## Abstract

We performed a serological, clinical, and parasitological follow-up of a dog population in an endemic area of American Visceral Leishmaniasis estimated by indirect immunofluorescent assay (IFA) and western blot (WB). After twelve months, the results obtained from IFA demonstrated that 50% were seropositive and two serological profiles were observed: the first one ranging from 1/40 to 1/80 and the second ≥1/160. By WB, it was observed that the same percentage and sera from positive dogs presented the recognition of the peptides of 29 and 32 kDa up to 8 months before IFA serum conversion. Among the positive dogs, all the sera from symptomatic ones with tissue parasitism recognized the peptide of 68.5 kDa. Our results suggest the need of modifications in the control measures regarding the elimination of the dogs. They also corroborate the high sensitivity and specificity of western blot in the diagnosis of canine leishmaniasis, suggesting the possibility of its association with IFA.

## 1. Introduction

Leishmaniasis, a disease caused by several species of a protozoa of the genus Leishmania (Kinetoplatida: Trypanosomatidae), is endemic in 88 countries [1] with epidemiological peculiarities according to factors such as human activity, parasite subpopulation, and wild and domestic sand fly fauna composition, among others. In the American continent, the disease, named American Visceral Leishmaniasis, occurs in the tropical and subtropical regions. In Brazil, besides being a rural zoonosis, Visceral Leishmaniasis is becoming a peri-urban and even urban zooanthroponosis [2].

Both domestic and wild canids are described as the most important reservoirs of *Leishmania infantum chagasi* [3, 4]. Canine leishmaniasis is a chronic infection that should be considered viscerocutaneous and present a more severe prognosis than human disease. Nevertheless, like in humans, dogs can display an asymptomatic form of the infection or may even evolve to resolution [5]. Symptoms of the overt form of the disease are nonspecific and include fever, weight loss, renal failure, lethargy, onychogryphosis, generalized lymphadenomegaly, skin, and ocular lesions [6].

In Brazil, the leishmaniasis control measures include the elimination of seropositive dogs based on indirect immunofluorescent assay (IFA). The culling of dogs based only on serological titers ≥1/40 may result in an undesirable outcome, because like in humans, dogs may control infection by *L. infantum* depending on the competence of their cellular immune response. Elimination of seropositive dogs that already controlled the infection will probably result in replacement by another susceptible animal, a new potential source of infection for men [7].

In the Old World, several studies have suggested the western blot technique as a very sensitive and precocious serological tool for the diagnosis of visceral leishmaniasis [8, 9]. Differently, in the New World there are scarce reports regarding the use of that technique, and few studies accomplished in Brazil corroborate the western blot as a reliable diagnostic assay. De Paula et al. [10] have demonstrated that the peptides of 29 and 32 kDa were recognized precociously by *Leishmania (i.) chagasi* parasitized dogs.

In the present study we reappraise the issue in dogs from an endemic peri-urban area in Rio de Janeiro state, where eleven human cases of visceral leishmaniasis have been detected, 25% of the cats [11] and 29% of the opossums [12] were seropositives, and* Lutzomyia longipalpis* represented 19% of the total sand fly population.

The purpose of this study was to perform a serological, clinical, and parasitological follow-up of naturally infected dogs in an endemic area of AVL, to analyze the kinetics of the infections by comparing the IFA and WB results and attempting to identify potential prognostic markers.

## 2. Materials and Methods

### 2.1. The Studied Area

Barra de Guaratiba is a strip of land localized between the Atlantic Ocean and the mountains of Pedra Branca, a massif belonging to Serra do Mar in Rio de Janeiro State. The climate is tropical, with an annual average temperature of 27.5°C and rainfall of 1400 mm. Secondary Atlantic Forest altered by anthropic action covers the mountain slopes.

The measures implemented by control program of AVL in this area included culling of serumpositive dogs (IFA on blood eluate) and spraying houses and annexes to control the adult forms of the vectors.

### 2.2. Dog Populations

At the beginning of the study there were 197 dogs. All the animals were monitored through visits to their home places from an endemic area in the municipality of Rio de Janeiro, Brazil and tested by an Immunofluorescence and western blot assays for the presence of specific antibodies. A great number of dogs were lost during the follow-up, because of culling by the control program, death by natural causes, and the relocation of the animals in other areas by the owners. From this initial sample, 60 remaining dogs were followed up every two months, during one year, as follows: months 2, 4, 6, 8, 10, and 12 correspond, respectively, to February, April, June, August, October, and December. Despite the control program, all the serumpositive dogs among those 60 remained in the area during the follow-up.

Dog population was composed of domestic dogs with little or no access to veterinary care. This work was carried out in accordance with the ethical principles of animal experimentation and it was approved by the Ethical Committee on the Use of Animals (CEUA) of Fundação Oswaldo Cruz (FIOCRUZ), Rio de Janeiro, Brazil.

The clinical evaluation that included palpation of lymph nodes, and an appreciation of the general health status of the animal, was carried out at the time of blood collection.

Blood was collected by puncture of the cephalic vein. Blood samples were allowed to clot and serum was separated by centrifugation (10 min/30.000 ×g) and stored at −20°C until use. 

None of the animals involved in our study were vaccinated.

### 2.3. IFA

The assay was carried out according to the procedures previously described by Camargo [13]. Serum dilutions ranging from 1 : 10 to 1 : 2560 were tested and as cut-off of the positive reaction titers a dilution of 1 : 40 was adopted, established by the Brazilian Ministry of Health.

### 2.4. Western Blot

Western blot analysis was carried out according to Aisa et al. [8]. Briefly, antigen was obtained from the protease inhibitor (Phenanthroline 10 mM, Phenylmethanesulfonyl fluoride 0.1 M, Iodoacetamide 0.2 M) containing soluble phase (39.191 ×g/30 minutes) of lysed (minibomb cell disruption chamber) *L. chagasi* strain L-579 promastigotes, separated on 12.5% polyacrylamide gels and blotted onto nitrocellulose paper (NCP). Sera diluted 1/50 were incubated with a strip of NCP overnight at 4°C. Bound immunoglobulins were detected by incubation with rabbit antidog IgG conjugated to horseradish peroxidase (Sigma, St Louis, MO) and developed with H_2_O_2_ (0,06% v/v) and diaminobenzidine (25% w/v, Sigma, St Louis, MO).

### 2.5. Parasitological Follow-Up and Necropsy of the Dogs

Parasitological follow-up: enlarged poplyteal lymph nodes observed in four of the 60 dogs were punctured by using Vacuntainer tubes containing NNN medium, with a Schneider's complete medium (Sigma, St Louis, MO) overlay that included 20% of fetal calf serum. The tubes were kept at 27°C and examined every five days during two months.

Necropsy: four of the 60 dogs were killed with an overdose of Thiopental sodium (10–25 mg/kg, IV) and potassium chloride (100 mg/kg, IV). At necropsy, Giemsa-stained imprints and smears were made of healthy ear and abdominal skin, liver, spleen, lymph nodes, and ascitic liquid and examined for the presence of amastigotes.

### 2.6. PCR

For DNA isolation and PCR analysis, dogs' spleen samples were mixed with a solution of Guanidine-EDTA in a proportion of 1 : 1 and after 24 hours the mixture was boiled for 1 minute and kept at 4°C up to DNA extraction. Total DNA was also obtained from cultured *L. *(*L*) *chagasi* promastigotes (MHOM/BR/1974/PP75) used as a positive control. PCR was carried out according to Passos et al. [14].

### 2.7. Dot Blot Analysis to Detect *Leishmania chagasi* Infection

The amplified products were also analyzed by dot blot hybridization as described by Francino et al. [15].

## 3. Results

### 3.1. IFA

In the beginning of the follow-up, 41.7% (25/60) of the dogs were seropositive, they were asymptomatic, and serological titers ranged from 1 : 40 to 1 : 640 (Table 1), in spite of the surveillance performed by the Brazilian Ministry of Health in the study area.

One year later, at the end of the study, 50% (30/60) of the animals were seropositive, so, the incidence of seroconversion was 8.3% (5/60) during the follow-up. The seroconversions were observed, respectively, on month 4 in 2 dogs, month 6 in 2 dogs, and month 12 in 1 dog (Table 1).

After twelve months two serological profiles were observed: the first one, ranging from 1/40 to 1/80, composed of 16 animals that stayed with low serological titers during the whole year and they did not present any symptoms; the second, with serological titers ≥1/160, composed of 14 animals, with a tendency to present higher serological titers and some dogs showing symptoms and tissular parasitism.

### 3.2. Clinical and Infection Status of Dogs

After one year, five of the 14 dogs belonging profile 2 displayed symptoms that appeared between 8 and 12 months and all of them had IFA titers ≥1/320. In all the five dogs were observed apathy and weight loss. Hepatosplenomegaly was observed in 2 animals, discrete lymphadenopathy in 2 animals, onychogryphosis in 1 animal, and ascitis in 1 animal (Table 2). One of the dogs died and the others were euthanasied and necropsied.

### 3.3. Western Blot Analyses

In the results obtained by western blot during the follow-up, the profile of antigenic recognition did not show any difference, except for the dogs that seroconverted that presented an increase of the number of bands and the intensity of its recognition.

**Figure 1:**
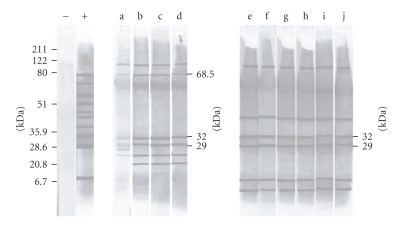
Antigenic recognition by western blot from sera of naturally infected dogs with *L.(i.) chagasi*. Lane (−): serum from serumnegative (IFA) dog. Lanes (+): serum from serumpositive (IFA 1/1280) dog. Lane a–d: serum from dog 18, that seroconverted to *Leishmania (i.) chagasi* (IFA) during follow-up. Lane a: IFA negative, western blot positive (bands 68.5, 32 and 29 kDa); b–d follow-up dog 18: IFA titers 1/1280 from month 2 of the follow-up onwards. Lane e–j: serum sample from dog 30 asymptomatic, IFA positive (1/640), and western blot positive (bands 32 and 29 kDa) at all six sample collections points.

**Figure 2:**
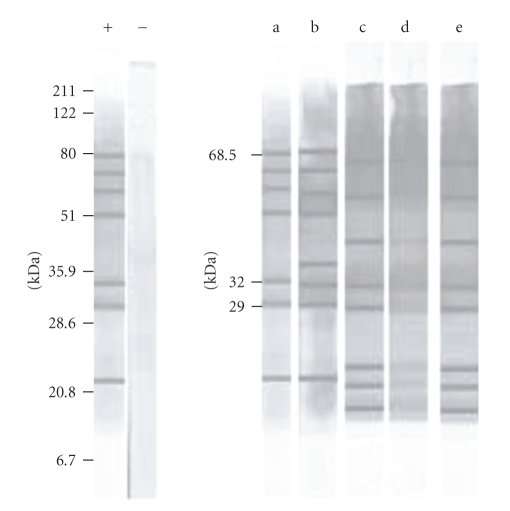
Antigenic recognition by western blot from sera of symptomatic and asymptomatic dogs with *L. (i.) chagasi. *Antigen recognition of *Leishmania (i.) chagasi* by serum from naturally infected dogs. Lane (−): serum from serumnegative (IFA) dog. Lane (+): serum from serumpositive (IFA 1/1280) dog. Lanes a and b: IFA seropositive symptomatic dogs. Lanes c, d, and e: IFA seropositive asymptomatic dogs.

It was observed a high seroprevalence (50%) with a strong and homogeneous pattern of antigenic recognition. The recognized polypeptide fractions were in the range of 4 to 220 kDa and all animals with confirmed infection systematically recognized peptide bands of 29 and 32 kDa (Figure 1). Only the sera from the 5 symptomatic dogs recognized the band of 68.5 besides those of 29 and 32 (Figures 1 and 2) and all those animals presented serological titers ≥1/320.

Sera from noninfected dogs from endemic and nonendemic areas displayed equivalent recognition patterns that included weak recognition of antigens of 37, 42.5, and 71.5 kDa. The thirty negative sera weakly recognized a maximum of five peptide fractions, not including the peptides 29, 32, or 68.5 kDa.

### 3.4. Parasitological Evaluation

In all the four animals that were necropsied, amastigotes were found in liver, spleen, skin, and lymph node imprints (Table 2).

In one of those animals, it was observed ascitis and from this dog, several liters of ascitic liquid were punctured intensely parasitized. The serological titers from the four animals that were necropsied were, respectively, 1/320 in one animal and 1/1280 in 3 animals. Two dogs with serological of 1/1280 were intensely parasitized.

Puncture of enlarged poplyteal lymph nodes from four out of 30 seropositive dogs was all negatives in attempt to isolate *Leishmania.*

### 3.5. PCR and Dot Blot Analysis to Detect *Leishmania (Leishmania) chagasi* Infection

All samples studied were realized in duplicate. In the visualization of the amplification products in agarose gel of two dogs samples, both were positives, amplifying the product of 120 pb, corresponding to the conserved kDNA region of *Leishmania sp*.

**Figure 3:**
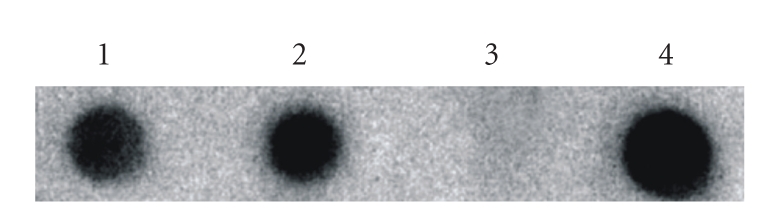
Hybridization with specific probe for *Leishmania infantum chagasi*. Amplified products from specific primers for the *Leishmania* (120 pb). (1) and (2) dogs positive samples, (3) negative sample, (4) positive* Leishmania (i.) chagasi* control. All samples studied were realized in duplicate.

After hybridization of this amplified product with an specific probe for *L. infantum*, the positive samples were confirmed as *Leishmania (i.) chagasi* and all negative samples maintained negative (Figure 3).

## 4. Discussion

In canine visceral leishmaniasis, low levels of antibodies production are related with low parasitemia, conversely, high antibody production has been associated with clinical disease and a best chance of parasite detection [7, 16, 17].

In the present study, we observed the occurrence of two serological profiles after one year of follow-up of a canine population by IFA. The animals of profile one, that presented low serological titers during the whole year, probably were either in the early phases of infection or controlled it in a similar mechanism as described in *Leishmania* Infantum [7, 18]. On the other hand all animals of profile two showed an increase on serological titers, some of them reaching 1/1280, suggesting that probably these animals lost their ability in controlling the infection, presenting within a few weeks symptoms and high number of parasites.

It has been proposed as an effective control measure, the culling of infective dogs with titer of ≥1/40 [19, 20]; however, some studies argue against using this measure [21, 22]. Our results suggest that in case of the adoption of control measures that include the retreat of the serumpositive dogs, the cut-off (1/40) must be maintained as the standard of positivity. Nevertheless, only the animals with titers of ≥1/160 that belong to profile two should be eliminated. It is likely that those animals that show high serological titers and tissular parasitism are more infective for sand flies because dog infectiousness and the proportion of sand flies infection were already suggested to be strongly correlated with anti-*Leishmania* antibody levels and intensity of skin disease [4, 20, 23].

In relation to the animals belonging to profile 1, they should still be monitored, because those dogs probably may reflect different clinicalepidemiological situation, such as early phases of infection or natural resistance of the host. 

In a longitudinal study performed on 43 beagle dogs exposed to three transmission seasons (2002 to 2004) of Mediterranean Leishmaniasis, the results observed by the authors corroborate with our data at least in part, because they identified as with patent infection, animals with serological titers ≥1 : 160 and tissular parasitism, presenting symptoms or not. On the other hand, dogs were considered with subpatent infection, when they present titers <1 : 160 without tissular detectable parasitism [24].

Moreover, culling animals that do not show signs of disease often results in owners hiding their animals, preventing them even to be examined and tested and consequently leading to the persistence of parasitized dogs.

Western blot assay has been considered as a highly specific and precocious method for the diagnosis of canine american visceral leishmaniasis because parasitic peptide fractions of 29 and 32 kDa were associated with occurrence of *Leishmania* infection in dogs [10]. Our results corroborate with those observations because the sera of all the infected dogs recognized such peptide fractions of 29 and 32 kDa up to 8 months before IFA serum conversion, although it has not implied in a prognosis for the outcome of disease.

The recognition of the 68.5 kDa band could be of prognostic value; indeed, only sera of the 5 symptomatic dogs reacted with this fraction while none of the sera from 25 asymptomatic dogs showed recognition (Figure 2). The same results were observed among sera of 12 symptomatic dogs in a population of 42 animals from another area not included in this study (data not shown).

According to Carrera et al. [25] in experimentally infected dogs with *L. infantum*, the recognition of some antigens by the sera of the animals with the worse clinical evolution was associated with active amastigote multiplication. We think the recognition of the 68.5 kDa peptide only by the sera of symptomatic dogs could represent a similar mechanism. 

Regarding the symptoms, in our study, it was not observed any sign that could be described as characteristic of leishmaniasis, because negative dogs also displayed the same symptoms as the positive ones, with the difference that in the negative dogs the symptoms tended to revert. Weight loss was the most frequent symptom observed among both the positive and negative dogs. In none of the animals skin lesions were found and it is worthwhile to point out that all the symptomatic animals presented amastigotes in skin of the ear and abdomen. Those observations demonstrated that dogs even without skin lesions, can represent an important infection source for sand flies.

Lymph nodes culture as also observed by others authors [26, 27] showed not to be a useful diagnostic method, it was not possible to isolate the parasite from dogs even with amastigotes in ear skin, liver, and spleen imprints, probably because of culture contamination occurred in function of the inadequate field conditions.

## Figures and Tables

**Table 1:** Serological and clinical follow-up of *Leishmania (i.) chagasi* infection in dogs. The correlation between serological titers, western blot, and symptoms in dogs, every two months during one year of follow-up. All sera from positive dogs recognized peptide of 29 and 32 kDa and sera from symptomatic dogs recognized peptide of 68.5 kDa. (−): negatives; (Prof): profile; (IFA): indirect immunofluorescent assay; (WB): western blot; (S): symptoms; (∗) dogs that seroconverted; (†) dogs died; (‡) dogs killed.

	Dogs *n*°	Month 2			Month 4			Month 6			Month 8			Month 10			Month 12		
		IFA	WB 68.5	S	IFA	WB 68.5	S	IFA	WB 68.5	S	IFA	WB 68.5	S	IFA	WB 68.5	S	IFA	WB 68.5	S
Prof 1	1–13	40	−	−	40	−	−	40	−	−	40	−	−	40	−	−	40	−	−
	14-15	80	−	−	80	−	−	80	−	−	80	−	−	80	−	−	80	−	−
	16*	−	−	−	40	−	−	40	−	−	40	−	−	40	−	−	40	−	−

Total	16																		

Prof 2	17*	−	−	−	−	−	−	40	−	−	40	−	−	160	−	−	640	−	−
	18*	−	+	−	160	+	−	1280	+	−	1280^‡^	+	+						
	19*	−	−	−	−	−	−	−	−	−	−	−	−	−	−	−	160	−	−
	20*	−	−	−	−	−	−	40	−	−	160	−	−	160	−	−	320	−	−
	21	40	+	−	80	+	−	40	+	−	160	+	−	160	+	−	320^‡^	+	+
	22	40	−	−	160	−	−	160	−	−	640	−	−	640	−	−	640	−	−
	23	40	−	−	160	−	−	1280	−	−	640	−	−	640	−	−	640	−	−
	24	80	−	−	80	−	−	80	−	−	640	−	−	320	−	−	640	−	−
	25	80	−	−	80	−	−	160	−	−	320	−	−	320	−	−	640	−	−
	26	80	+	−	160	+	−	640	+	−	1280	+	+	1280^‡^	+	+			
	27	160	+	−	320	+	−	160	+	−	320	+	+	320^†^	+	+			
	28	160	+	−	80	+	−	160	+	−	320	+	−	1280^‡^	+	+			
	29	320^†^	−	−															
	30	640	−	−	640	−	−	1280	−	−	1280	−	−	1280	−	−	640	−	−

Total	14																		

**Table 2:** Serological, clinical, and parasitological profiles of *Leishmania (i.) chagasi* infection in dogs. Description of the symptoms of the positive dogs and correlation with IFA, WB, and presence of parasites.

Dogs *n*°	Clinical status	IFA	WB		Presence of parasites					
			29/32	68.5	Ear skin	Abdominal skin	liver	spleen	Lymph nodes	Ascitic liquid
18	Apathy, weight loss, hepatosplenomegaly onychogryphosis	1280	+	+	++	++	++	++	++	−
21	Apathy, weight loss, lymphadenopathy	320	+	+	+	+	+	+	+	−
26	Apathy, weight loss,	1280	+	+	+	+	+	+	+	−
28	Apathy, weight loss, hepatosplenomegaly lymphadenopath, ascitis.	1280	+	+	++	++	++	++	++	++

IFA: indirect immunofluorescent assay; WB: western blot.
